# Normative Data of Thyroid Volume-Ultrasonographic Evaluation of 422 Subjects Aged 0-55 Years

**DOI:** 10.4274/jcrpe.1818

**Published:** 2015-06-03

**Authors:** Ömer Aydıner, Elif Karakoç Aydıner, İhsan Akpınar, Serap Turan, Abdullah Bereket

**Affiliations:** 1 Anadolu Health Center, Clinic of Radiology, İstanbul, Turkey; 2 Marmara University Faculty of Medicine, Department of Pediatrics, Division of Pediatric Allergy and Immunology, İstanbul, Turkey; 3 Marmara University Faculty of Medicine, Department of Radiology, İstanbul, Turkey; 4 Marmara University Faculty of Medicine, Department of Pediatric Endocrinology, İstanbul, Turkey

**Keywords:** child, normative data, thyroid volume, ultrasonography

## Abstract

**Objective:**

To establish local normative data of thyroid volume assessed by ultrasonography in subjects aged 0-55 years living in İstanbul, Turkey.

**Methods:**

Subjects without any known history of thyroid disease, of major surgery and/or chronic disease were enrolled in the study and evaluated by physical examination and thyroid ultrasonography. Thyroid gland and isthmus at usual location, each lateral lobe volume with three dimensions, ectopic thyroid tissue and echogenicity of the gland were assessed.

**Results:**

Initially, 494 subjects were enrolled in the study. Subjects showing heterogeneous thyroid parenchyma (n=21) and/or nodule (n=51) in ultrasonography were excluded. Final analysis covered 422 subjects (216 males, 206 females). Thyroid volume was found to significantly correlate with height, weight, age and body surface area (r=0.661, r=0.712, r=0.772 and r=0.779, respectively; p<0.0001 for all). These correlations were even stronger in subjects younger than 18 years (r=0.758, r=0.800, r=0.815 and r=0.802, respectively; p<0.0001 for all).

**Conclusion:**

The study provides updated reference norms for thyroid volume in Turkish subjects which can be used in the diagnosis and follow-up of patients with thyroid diseases.

## INTRODUCTION

Evaluation of thyroid size is important in the diagnosis and management of thyroid disorders and iodine deficiency disorders. Thyroid size can be estimated manually, but this method is open to subjectivity and can be difficult especially in young children. Ultrasonography is a non-invasive method which provides a three-dimensional measurement of the thyroid gland and is currently the method of choice for evaluating thyroid size. Age-and sex-related normative data obtained from healthy individuals is a prerequisite for diagnosing thyroid enlargement (goiter) or thyroid hypoplasia. Furthermore, evaluation of cystic and solid lesions, three dimensions of each lobe, thickness of the isthmus and echogenicity of the gland are all possible by ultrasonography.

Normative data of thyroid volumes showing variations with age, regional factors and iodine status of the population have been reported in different populations (1,2,3,4). At present, only limited data are available for the Turkish population on thyroid volumes and no study to date included subjects from the newborn period up to 55 years of age. Furthermore, iodine supplementation programs conducted during the past 15 years in Turkey have changed iodine status and have created a need for new updated reference data for thyroid volumes. In the present paper, we aimed to establish local normative data of thyroid volume assessed by ultrasonography in subjects living in İstanbul.

## METHODS

### 

The Ethics Committee of the Marmara University Medical School approved the study (B.30.2.MAR.0.01.00.02/AEK-171). Written informed consent was obtained from the parents of each child and from the subject if older than 18 years. Healthy subjects aged 0-55 years were enrolled. Body weight and height were measured with an infantometer (Seca 210, Hamburg, Germany) in subjects aged less than 2 years and with a Harpenden stadiometer in those older than 2 years. Thyroid palpation was performed by the same physician (EKA).

Subjects having no known thyroid disease, surgery and chronic disease were included in the study, while those having a palpable thyroid gland in the clinical examination and those showing nodules and/or heterogeneous parenchyma in thyroid ultrasonography were excluded.

Thyroid ultrasonography was performed by two experienced radiologists (OA and IA), using gray-scale ultrasonography with a 7 MHz linear probe (GE Medical System MR Logic 700, Milwaukee, Wisconsin) and pre-warmed gel. Figures for intra- and inter-observer variations were found to be statistically insignificant. The subjects were examined in the supine position with hyperextended neck. The sonogram was evaluated for presence or absence of thyroid gland and isthmus at their normal locations, for volume of each lateral lobe calculated by measuring three dimensions and for presence or absence of ectopic thyroid tissue and echogenicity of the gland in the anterior cervical area through the suprasternal area.

Thyroid volume was estimated using the following equations:

Thyroid volume (mL)={(R2 x R2 x R3 x 0.5)/1000}+{(L1 x L2x L3 x 0.5)/1000}

Body surface (m2)=√[height (cm) x weight (kg)/3600]

### Statistical Analyses

Statistical analyses were carried out using the Statistical Package for the Social Sciences (SPSS) program (Version 17.0; SPSS Inc., Chicago, IL, USA). Normality of distribution was tested for each parameter by one-sample Kolmogorov-Smirnov test. Parameters in this test were expressed as means and standard deviations. The relationships of thyroid volume with age, body weight, height and body surface were evaluated using the Pearson’s correlation test.

## RESULTS

The subjects (n=494) who met the inclusion criteria listed above were enrolled in the study and all were evaluated by physical examination and thyroid ultrasonograpy. Subjects showing heterogeneous thyroid parenchyma (n=21) and/or nodule (n=51) on ultrasonography were excluded from the study and statistical analyses. As a result, 422 subjects (216 male, 206 female) were included in the final analyses. Demographic and ultrasonographic data of the subjects for age groups are presented in Table 1. Thyroid volumes and the three dimensions of the right and left lobes and of the isthmus of each age group did not show statistically significant differences between males and females.

**Figure 1 A-D:**
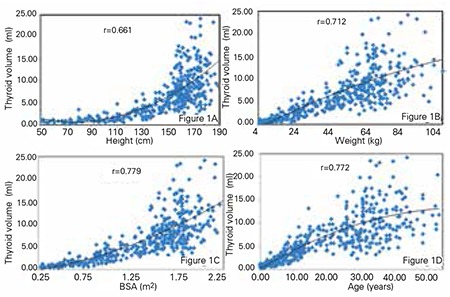
The correlations of thyroid volume-height in Figure 1A, thyroid volume-body weight in Figure 1B, thyroid volume-body surface area in Figure 1C and thyroid volume-age in Figure 1D are shown for all subjects enrolled into the study. Pearson’s correlation is expressed as ‘r’ coefficient at the top of the figure.

The correlations between thyroid volume and stature are shown in Figure 1A, between thyroid volume and body weight in Figure 1B, between thyroid volume and body surface area in Figure 1C and those between thyroid volume and chronological age in Figure 1D. Thyroid volume was found to positively correlate with height, weight, age and body surface area in all subjects (r=0.661, r=0.712, r=0.727 and r=0.779, respectively; p<0.0001 for all).

**Figure 2 A-D:**
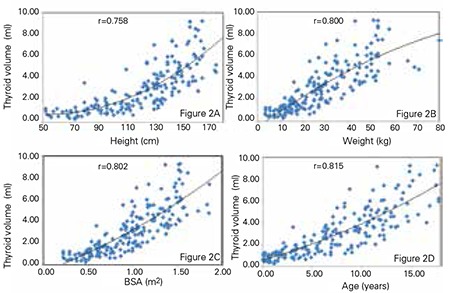
The correlations of thyroid volume-height in Figure 2A, thyroid volume-body weight in Figure 2B, thyroid volume-body surface area in Figure 2C and thyroid volume-age in Figure 2D are shown for subjects less than 18 years of age. Pearson’s correlation is expressed as ‘r’ coefficient at the top of the figure.

When subjects younger than 18 years of age were evaluated separately, thyroid volume was also found to positively correlate with height, weight, body surface area and age (r=0.758, r=0.800, r=0.802, r=0.815, respectively; p<0.0001 for all). These data are presented in Figure 2 A-D. On the other hand, in subjects older than 18 years of age, the correlations were as follows; between thyroid volume and body weight – r=0.260, between thyroid volume and statural height – r=0.269, between thyroid volume and body surface area – r=0.299 and between thyroid volume and chronological age – r=0.306 (p<0.0001 for all).

## DISCUSSION

The current study has provided normative data for thyroid volume at all ages, covering age groups from neonate up to 55 years. Thyroid volumes increased from birth to adulthood and the increase was most remarkable at pubertal ages. Body surface area was the strongest correlating factor with thyroid volume in the whole group, whereas age was the strongest correlate in children.

Normative values of thyroid size have been studied previously throughout the world (3,5,6). These data showed a wide range of differences among their results, most likely due to regional and ethnic factors as well as to the iodine status of the population. Zimmermann et al (7) reported reference values for thyroid volume by ultrasound in iodine-sufficient schoolchildren from various nationalities, re-emphasizing the importance of establishing local data for each country. Furthermore, regional differences in thyroid size even in the same country have been reported both from Italy and Turkey for the adult population (8,9,10,11). Thus, it is obvious that normative data on thyroid size needs to be updated. The current study provides reference data on thyroid volume in a population living in İstanbul, Turkey and aged from birth to 55 years.

The World Health Organization (WHO) reported a 20% reduction in thyroid volume in year 2004 when compared to 1997, even in populations with the same ethnicity and living in the same geographical areas (4,7). This finding could be interpreted to indicate application of effective iodine supplementation policies throughout Europe. Accordingly, Szybinski et al (3) from Poland reported lower thyroid volumes in 2012 compared to 1997, but higher volumes compared to the 2004 WHO data. In Turkey, thyroid volumes of healthy newborns were evaluated by Kurtoglu et al (12,13) in 1994 and 2004 and their findings showed a decline in the mean volume from 1.2 to 0.8 mL. In this present study also, thyroid volumes were found to be lower than those reported from Turkey by Taş et al in 2002 (1). These findings indicate that there is a need for renewal of thyroid normative data also in the coming years in Turkey.

Thyroid volume was reported to be correlated with weight, height, body surface area and age in children aged 6-11 years; the highest correlation was found between thyroid volume and age (11). The current study also confirmed this finding in subjects younger than 18 years. However, in one other study on Turkish children aged 0-16 years from an endemic iodine-deficient area, weight was reported as the factor showing the highest correlation with thyroid volume (1).

Several studies to establish reference data by thyroid ultrasonograhy in adults have also been conducted. In an iodine-sufficient population, an age-related increase in thyroid gland volume for both sexes was reported in healthy adults (14). In addition, Gomez et al (15) showed that body surface area accounts for much of the variation of thyroid volume and that male had higher volumes than females. In the current study, we found no differences relating to gender. Thyroid volume in adults was also found to be related to age with an r value of 0.309, which indicates a rather weak correlation.

To our knowledge, this is the first study reporting normative data on thyroid volume covering a wide age range in Turkey. Although the data are based on a population residing in a limited region of the country, İstanbul is the largest city of the country with a high immigration rate from all over the country. Thus, we believe that our data can serve as a reference for other regions of the country where regional data are not available. On the other hand, we realize that sample size is a limitation of the current study and we believe that there is a need for future studies on larger samples.

In conclusion, in view of a need to evaluate thyroid size in comparison with normative data obtained from age-matched subjects from the same population for the diagnosis of either goiter or hypoplasia/dysgenesis, we believe that this study provides an age-matched reference data covering a large age range on a population residing in İstanbul.

## Figures and Tables

**Table 1:**
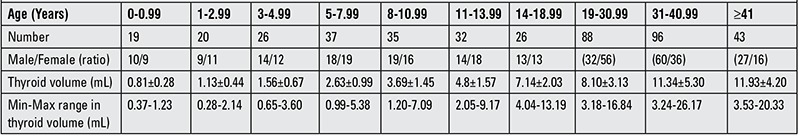
Demographic and ultrasonographic data of the subjects by age groups.
